# Multidimensional mechanics: Performance mapping of natural biological systems using permutated radar charts

**DOI:** 10.1371/journal.pone.0204309

**Published:** 2018-09-28

**Authors:** Michael M. Porter, Pooya Niksiar

**Affiliations:** Department of Mechanical Engineering, Clemson University, Clemson, SC, Untied States of America; Rush University Medical Center, UNITED STATES

## Abstract

Comparing the functional performance of biological systems often requires comparing multiple mechanical properties. Such analyses, however, are commonly presented using orthogonal plots that compare *N* ≤ 3 properties. Here, we develop a multidimensional visualization strategy using permutated radar charts (radial, multi-axis plots) to compare the relative performance distributions of mechanical systems on a single graphic across *N* ≥ 3 properties. Leveraging the fact that radar charts plot data in the form of closed polygonal profiles, we use shape descriptors for quantitative comparisons. We identify mechanical property-function correlations distinctive to rigid, flexible, and damage-tolerant biological materials in the form of structural ties, beams, shells, and foams. We also show that the microstructures of dentin, bone, tendon, skin, and cartilage dictate their tensile performance, exhibiting a trade-off between stiffness and extensibility. Lastly, we compare the feeding versus singing performance of Darwin’s finches to demonstrate the potential of radar charts for multidimensional comparisons beyond mechanics of materials.

## Introduction

**Fig 1:**
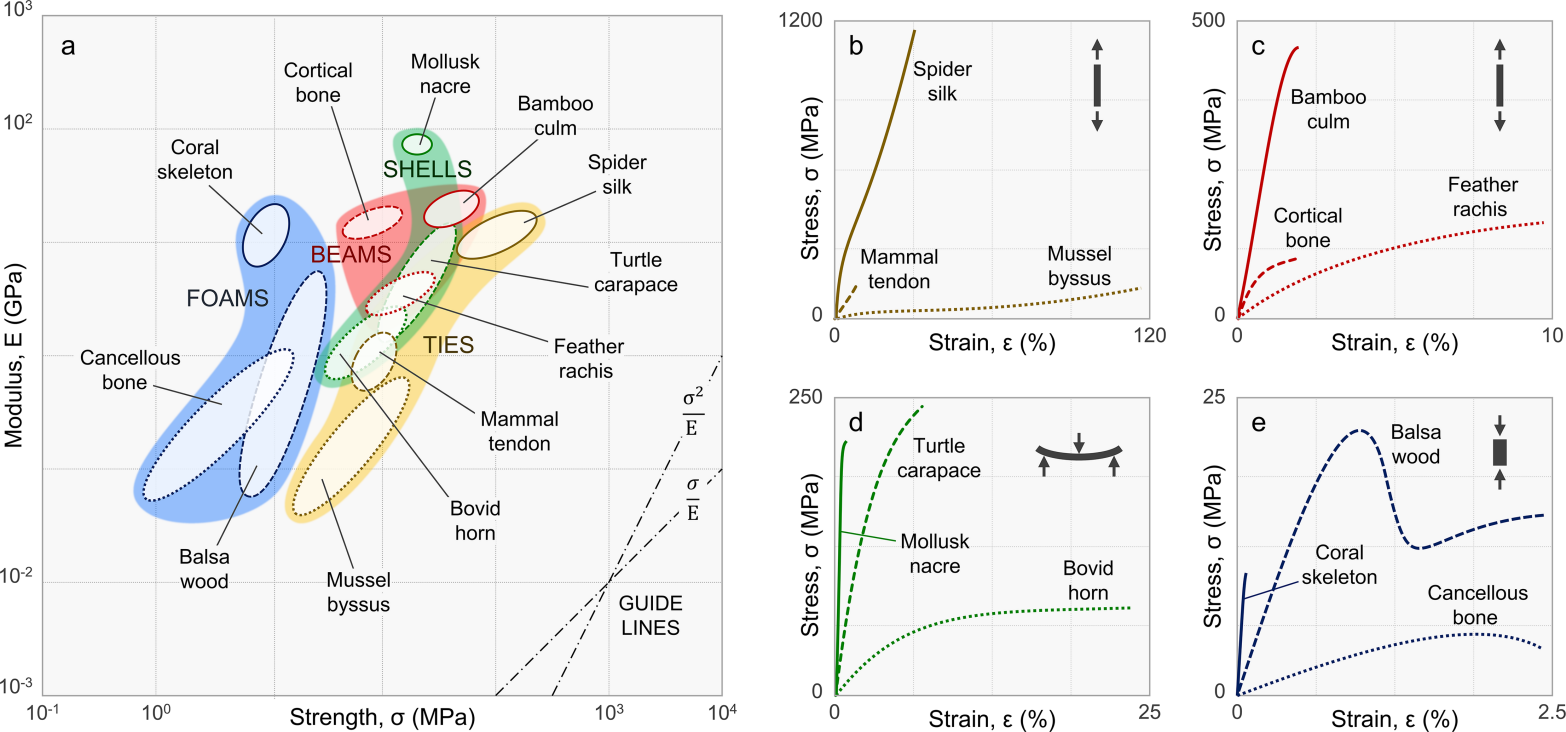
Mechanical properties of structural biological materials. (a) Mechanical property chart comparing the strength and modulus of twelve natural materials (see Box 1); (b-e) representative stress-strain curves of the four structural classes of materials. Notes: Guidelines shown in (a) are derived material indices for elastic hinges (*σ*/*E*) and springs (*σ*^2^/*E*) as defined by Ashby [4]. The icons in the upper-right corners of (b-e) indicate the modes of loading: tension, bending, or compression. Data used to create the figures are listed in S1–S6 Tables, compiled from numerous sources (see Supporting Information).

Natural biological materials often exhibit unprecedented combinations of multiple mechanical properties [1] and functional performance [2, 3]. Yet, comparisons are commonly displayed on two- or three-dimensional property charts [4], like the strength-modulus chart in Fig 1A. Such data is usually extracted from stress-strain (or force-displacement) curves, like those in Fig 1B–1E. Depending on the mode of loading (tension, compression, bending, etc.), a stress-strain plot contains a distinct set of multidimensional property data that describes a material’s behavior, including, for example, its stiffness, strength, toughness, resilience, and strain to failure. As a result, comparisons of different structures across different modes of loading typically involve a concurrent analysis of multiple orthogonal plots comparing *N* ≤ 3 properties [5]. Here, we introduce a relatively simple and accessible method using radar charts (radial, multi-axis plots—also called star, polar, wheel, spider, web, kiviat, or circular parallel coordinate charts) [6–8] to compare the multidimensional performance of mechanical systems across *N* ≥ 3 properties.

Unlike most other multidimensional data visualizations and reduction analyses [9–11], radar charts plot multiple dimensions on a single graphic in the form of closed polygonal profiles of definite size, position, and shape. Much like a probability distribution function, a profile plotted on a radar chart represents a system’s relative performance distribution measured across *N* ≥ 3 comparative properties, where each property is represented by an axis of the chart. Although radar charts have been criticized for their subjectivity in axis-sorting [12, 13], we suggest that the unique polygonal structure of their data provides a convenient platform for comparing mechanical systems. Similar to existing dimension ordering heuristics [14–17], we show that like properties (axes) are sorted together by maximizing the total area of the plotted profiles, creating a useful stage for multidimensional performance comparisons.

Following this permutation scheme, we use radar charts as tools to compare the multidimensional mechanics of several natural biological materials and structures. When a radar charts’ axes represent mechanical properties (modulus, strength, etc.), the relative performance distributions of the comparative systems can be characterized by the geometries of their property profiles. Using established techniques from pattern recognition [18–20], we show that it is possible to identify performance trade-offs, compare functional similarities, and quantify the relative multidimensional behaviors of mechanical systems via shape moments and other geometric descriptors. Specifically, we analyze the mechanical property distributions of several structural biological materials and collagenous tissues exhibiting a wide range of functionalities, whose different properties were compiled from literature. Agreeing with conventional wisdom, our analyses reveal clear trade-offs between stiffness versus strain to failure and several distinctive property-function correlations. We also demonstrate the extended potential of radar charts, beyond material comparisons, with a case study on Darwin’s finches, whose beaks show a distinct trade-off between feeding versus singing performance. Thus, we propose that the polygonal structure of data unique to radar charts permits the use of simple shape descriptors to compare the relative multidimensional mechanics of natural systems.

## Materials and methods

### Mechanical property data

A list of data source references is included in the Supporting Information. To present a fair comparison of mechanical property data, we searched the literature to generate six datasets of comparative mechanical properties, which were respectively reported using similar testing protocols and units (see S1–S6 Tables). For all material classes (ties, beams, shells, and foams) and collagenous tissues (dentin, bone, tendon, skin, and cartilage) we compiled available data on the elastic and shear moduli (E and G), strain to failure (ε), strength in tension, compression and flexure (σ_T_, σ_C_, and σ_F_), resilience (u_R_), toughness (u_T_ or K_IC_), damping loss factor (tan δ), hardness (H), and impact strength (IS). For Darwin’s finches, we compiled data from male species on their beaks’ maximum gape, base and tip bite forces, opening and closing velocities, and vocal deviations. In most cases, these data were taken from two or three (at most five) different studies. For each system, effort was made to collect as much data as possible from a single source or research group, such that data plotted on the radar charts would be as consistent and representative as possible. We also cross-checked multiple literature sources to verify accuracy of compiled datasets, which resulted in no missing data (except for some measures of density). The captions and notes of S1–S6 Tables provide additional information on the types of data sources and any unit conversion factors that were applied to the data, which are reported as averages, standard deviations, standard errors, and/or ranges. All plots, including radar charts, were generated using MS Excel 2016 (Microsoft, Redmond, WA) and MATLAB R2018a (MathWorks, Natick, MA).

### Axis sorting

**Fig 2:**
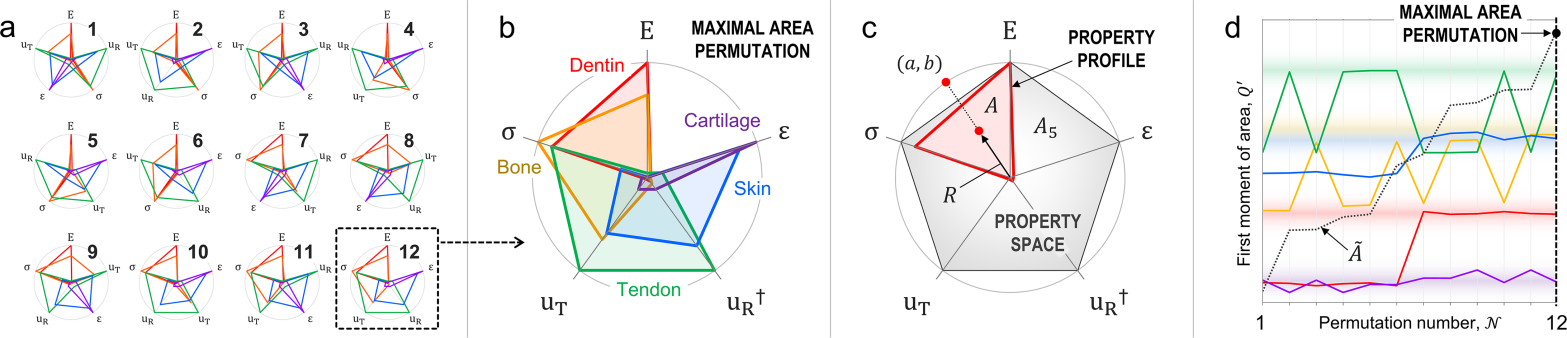
Methods of axis-sorting and shape moments using radar charts. (a) All possible permutations of the *N* = 5 mechanical properties of the collagenous tissues dataset, where the instance resulting in a maximal total area (12) is boxed with a dashed line. (b) The “permutated radar chart” yielding a maximal total area used for shape descriptor analyses. (c) The property profile of dentin (red), illustrating its area (*A*), centroidal distance (*R*), and the *x*,*y*-coordinates of point (*a*,*b*) located at the intersection of a vector passing through the profile’s centroid and the perimeter of a unit circle; the area of the property space (grey) of a regular pentagon is *A*_5_ ≈ 2.4. (d) The relative first moment of area *Q*′ about the outer limits of the property space (*a*,*b*) for the five materials across all 12 permutations; notice that the maximum values for *Q*′ are nearly equivalent to those of the maximal area permutation. Legend: dentin = red; bone = yellow; tendon = green; skin = blue; cartilage = purple; total permutation area ($\tilde{A}$) = black dashed line (right axis).

Mechanical properties (axes) were sorted using a custom MATLAB routine, which searched for the appropriate permutation of axes resulting in maximal total area. To explain the permutation scheme, we use for an example the collagenous tissues dataset (S5 Table). Fig 2 outlines the process. MATLAB code is available in the Supporting Information. For a dataset of *N* properties (axes), the number of circular permutations is:

$$N=\frac{(N-1)!}{2}.$$ (1)

Fig 2A shows the 12 possible permutations for the collagenous tissues dataset, where the vertices of each profile are mean value coordinates [21] of each property normalized by its maximum:

$$p_{ij}={\bar{P}}_{ij}/\max{\bar{P}}_{i}$$ (2)

where ${\bar{P}}_{ij}$ and ${\bar{P}}_{i}$ are the averaged properties (*i* = 1, 2,…, *N*) of each system (*j* = 1, 2,…, *n*) and the total dataset, respectively. The profile areas of each permutation were calculated using the MATLAB function: polyarea(X,Y); alternatively, they can be calculated as:

$$A_{j}=\frac{1}{2}{\left(\left|\begin{array}{cc} x_{1} & x_{2}\\ y_{1} & y_{2} \end{array}|+|\begin{array}{cc} x_{2} & x_{3}\\ y_{2} & y_{3} \end{array}|+\cdots +|\begin{array}{cc} x_{N} & x_{1}\\ y_{N} & y_{1} \end{array}\right|\right)}_{j}$$ (3)

where the vertices *p*_*ij*_ are expressed in terms of Cartesian coordinates (*x*_*i*_,*y*_*i*_)_*j*_. Then, the targeted sequence of axes for shape analyses is the one that yields a maximal total area—i.e., the maximum sum of polygonal areas corresponding to each system of a comparative dataset:

$$\tilde{A}=\max\;\sum_{j=1}^{n}A_{j}.$$ (4)

Fig 2B shows this maximal area permutation for the collagen dataset. Throughout the paper, we refer to this sequence as the “permutated radar chart”. Geometric descriptors (Jaccard indices, shape moments, etc.) used to compare property profiles were calculated from this instance. For exploratory purposes, we ran exhaustive searches on all permutations, where *n* ≤ 8 systems and *N* ≤ 6 properties for all datasets (S1–S6 Tables). However, the number of operations necessary to find the maximal area permutation, for larger datasets (*N* ≥ 4), can be reduced by employing various heuristics, such as a greedy successive addition of dimensions [16].

### Jaccard indices

MATLAB was used to find the Jaccard index [22] for each pair of profiles on the permutated radar charts, which was calculated as the relative intersection over union:

$$J=\frac{\left|A\cap B\right|}{\left|A\cup B\right|}=\frac{\left|A\cap B\right|}{\left|A|+|B|-|A\cap B\right|}$$ (5)

where *A* and *B* are the enclosed areas of two profiles, and their intersections were evaluated by MATLAB using the function: areaintersection.m (Paul Koprowski, 2007).

### Shape moments

Additional comparative metrics can be defined by the profiles’ geometric shape moments [20]:

$$m_{\alpha \beta }=\int \int x^{\alpha }y^{\beta }\;f(x,y)\;dx\;dy$$ (6)

where (*α* + *β*) defines the order of the moment of a polyline *f*(*x*,*y*) that describes the size, position, and shape of each profile. Because radar chart data are structured in the form of closed polygons, their shape moments can be calculated following a procedure by Leu [23], which decomposes closed polygons into multiple triangles of vertices (0,0), (*x*_*i*_,*y*_*i*_) and (*x*_*i*+1_,*y*_*i*+1_):

$$m_{pq}=\sum_{i=1}^{N}m_{pq,T_{i}}∙sign(i)$$ (7)

where sign(*i*) is the sign of triangle *T*_*i*_, which is positive when $\tan^{-1}\left(\frac{y_{i}}{x_{i}}\right)\geq \tan^{-1}\left(\frac{y_{i+1}}{x_{i+1}}\right)$ and negative otherwise. The first three lower-order moments describe a profile’s area and centroidal distance from the origin: *A* = *m*_00_ and $R=\sqrt{{\left(\frac{m_{10}}{m_{00}}\right)}^{2}+{\left(\frac{m_{01}}{m_{00}}\right)}^{2}}$. The moments about a point (*a*,*b*) located at the intersection of a vector passing through the profile’s centroid and the perimeter of a unit circle centered at the origin is defined as (see Fig 2C):

$$M_{pq}=\int \int {\left(x-a\right)}^{p}({y-b)}^{q}f(x,y)dxdy$$ (8)

where (*a*,*b*) are defined as $a=m_{10}/\sqrt{m_{10}^{2}+m_{01}^{2}}$ and $b=m_{01}/\sqrt{m_{10}^{2}+m_{01}^{2}}$ when *m*_10_ and *m*_01_ are first-order shape moments (Eq 6). Accordingly, *M*_10_ and *M*_01_ can reduce to:

$$M_{10}=m_{10}\left(1-\frac{m_{00}}{\sqrt{m_{10}^{2}+m_{01}^{2}}}\right);$$ (9)

$$M_{01}=m_{01}\left(1-\frac{m_{00}}{\sqrt{m_{10}^{2}+m_{01}^{2}}}\right).$$ (10)

Then, the magnitude of the first moment of area about (*a*,*b*) can be expressed in terms of the profile area (*A*) and the Euclidean distance of its centroid from the origin (*R*):

$$Q=\sqrt{M_{10}^{2}+M_{01}^{2}}=A(1-R);$$ (11)

This metric is much like the moment of a physical quantity (in physics) or the mean of a distribution function (in statistics) [20]. By analogy, we define the relative multidimensional performance of a system as its profile’s normalized first moment of area relative to the boundary of the property space:

$$Q^{\prime }=\frac{Q}{Q_{N}}=\frac{A}{A_{N}}(1-R)$$ (12)

where *A*_*N*_ and *Q*_*N*_ are the zeroth and first moments of area about (*a*,*b*) of the property space, which is defined as a regular *N*-sided polygon of unit circumradius with its centroid at the origin, representing “maximal performance” with *p*_*i*_ = 1 across all *N* properties. In Fig 2C, *A*_5_ ≈ 2.4 is the area of a regular pentagon with a unit circumradius. Notably, the property space converges from a regular *N*-sided polygon where $A_{N}=\frac{1}{2}N\;\sin\left(2\pi /N\right)$ when *N* ≥ 3 to a unit circle where *A*_∞_ = *π* when *N* → ∞; although, higher-dimensional data (*N* ≫ 10) are likely not well represented using radar charts because of dimensional “crowding” and limitations on the information processing capacity of humans [24]. Albeit, axis-sorting could increase this capacity by clustering correlated dimensions. Finally, we calculated *Q*′ for all possible permutations of the different datasets. Comparing the maximum values of *Q*′ with those of the maximal area permutation (shaded regions in Fig 2D), we confirmed that the permutated radar chart provides a sufficient, relative approximation of the maximal metrics, and thus appropriately describes the relative multidimensional performance of the comparative mechanical systems discussed here.

### Other shape descriptors

In addition to Jaccard indices and shape moments, numerous other simple shape descriptors or combinations thereof (e.g., compactness, ratio of principle axes, circular or elliptical variance [18]) could be applied to compare radar chart data. To demonstrate, we compared the compactness of profiles corresponding to the dataset for Darwin’s finches (S6 Table). This metric is calculated as the perimetric ratio of a circle of equal area to that of a profile:

$$C=\frac{2\sqrt{\pi A}}{P}$$ (13)

where *A* and P are a profile’s area and perimeter, respectively.

## Results

### Property-function correlations of biological materials

**Fig 3:**
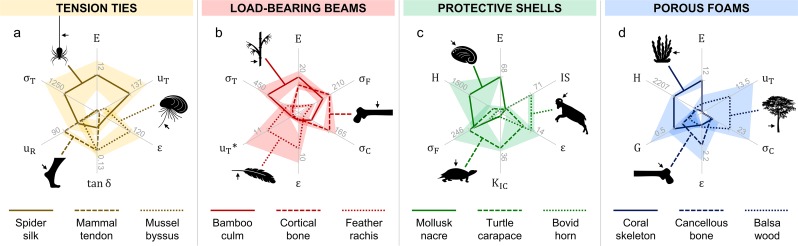
Multidimensional mechanics of structural biological materials. Normalized, permutated radar charts comparing four structural classes of natural materials (see Box 1). Legend (units): elastic and shear moduli, E and G (GPa); strain to failure, ε (%); tensile, compressive and flexural strength, σ_T_, σ_C_ and σ_F_ (MPa); toughness, u_T_ (MJ·m^-3^) or u_T_* (kJ·m^-2^); fracture toughness, K_IC_ (MPa·m^1/2^); resilience, u_R_ (%); damping loss factor, tan δ (no units); hardness, H (MPa); impact strength, IS (kJ·m^-2^). Notes: Data averages (lines) and standard deviations/errors/ranges (shaded regions) are listed in S1–S4 Tables, compiled from numerous sources (see Supporting Information); for reference, maximum values of each average property are displayed on the axes.

Structural biological materials are commonly classified by their mechanical properties [1], form and function [2, 3] (see Fig 1). Here, we compare four structural classes of natural biological materials: tension ties, load-bearing beams, protective shells, and porous foams. The selected materials were chosen for their representative, yet widely disparate functionalities. Their mechanical behaviors, form and function are briefly discussed in Box 1, with their normalized property profiles shown in Fig 3. For more details, readers are referred to the original data sources (see reference list in Supporting Information).

**Box 1. Structural biological materials. The general structure and function of four classes of biological materials: tension ties, load-bearing beams, protective shells, and porous foams (compare with Figs 1 and 3)**

***Tension ties***: *Structural elements typically in the form of thin fibers that carry tensile loads*. Fig 3A compares three types of natural tie materials: *spider silk* (dragline) is a tough, semi-crystalline fiber used for safety lines and the framing of spider webs [25]; *mammal tendon* is a resilient, partially-mineralized fiber that stores and releases energy for mobility and locomotion [26]; *mussel byssus* threads are gradient networks of highly-extensible fibers with abrasion-resistant coatings that the mollusks use to anchor onto wet (or dry) substrates [27].

***Load-bearing beams***: *Structural elements typically in the form of straight*, *often cylindrical beams*, *columns*, *or shafts that carry flexural moments and/or axial loads*. Fig 3B compares three types of natural beam materials: *bamboo culm* is a stiff, fibrous material with a hierarchical porosity gradient that supports the tall, hollow plants [28]; *cortical bone* is a strong, mineralized material organized into compact osteons that carry a multitude of skeletal moments and axial loads [29]; *feather rachis* is a resilient, sandwich composite of lightweight foam surrounded by a dense cortex that supports flexural moments during flight [30].

***Protective shells***: *Structural elements typically in the form of thin shells or coverings that protect against damage from abrasion*, *puncture*, *fracture*, *and/or impact*. Fig 3C compares three types of natural shell materials: *mollusk nacre* is a rigid, highly-mineralized material organized into brick-and-mortar microstructures that protect the soft-bodied mollusks from abrasion and puncture [31]; *turtle carapace* is a tough, fracture-resistant material composed of interlocking layered scutes that protect the animals from a variety of environmental threats and predators [32]; *bovid horn* is a resilient, energy-absorbent material that protects bovids, such as bighorn sheep, from impact during head-butting rituals and courtship [33].

***Porous foams***: *Structural elements typically in the form of scaffold-like foams that reduce weight and transfer flexural moments and/or axial loads*. Fig 3D compares three types of natural foam materials: *coral skeleton* is a rigid, highly-mineralized material that supports the organisms under variable ocean currents and protects them from predators [34]; *cancellous bone* is a lightweight, mineralized material organized into trabecular networks that redistribute stresses from cortical bone during load-bearing activities [29]; *balsa wood* is a lightweight, highly-porous material that supports the large, fast-growing trees [35].

As illustrated in Fig 3, the selected materials show a wide range of mechanical functionalities, depicted by their different performance profiles. Practically speaking, no one property is necessarily the best descriptor of a functional task or application. Instead, a combination of two or more properties is often necessary to describe a material’s functional performance. For example, the radar chart comparing tension ties in Fig 3A shows spider silk as the stiffest (E), strongest (σ_T_), and toughest (u_T_) fiber of the comparison. In contrast, mammalian tendons and mussel byssal threads exhibit similar damping indices (tan δ), but respectively high resilience (u_R_) and extensibility (ε) that distinguish their unique functions—i.e., tendons store and release energy; byssal threads absorb and dissipate energy. Hence, the sequence of axes defined on the permutated radar chart suggests that fiber stiffness, strength, and toughness are correlated with bearing high tensile loads, while fiber damping, resilience, and extensibility are correlated with the transfer and dissipation of strain energy (for this specific comparison). Likewise, the other three radar charts in Fig 3B–3D show some distinctive property correlations: for the beams, the elastic modulus (E) is correlated with all modes of strength (σ_T_, σ_C_, and σ_F_); for the shells and foams, the moduli (E and G) are correlated with hardness (H)—i.e., stiffer materials are generally stronger and harder. However, unlike tension ties, enhanced toughness (u_T_ and K_IC_), and strain to failure (ε), come at the cost of diminishing stiffness (E and G) in the beams, shells, and foams; and impact strength (IS) is correlated with strain to failure (ε).

As shown, the materials of each class that are more rigid, with large E, generally exhibit different/opposing property profiles than those that are more flexible, with large ε (see Fig 3). Damage-tolerant or tough materials, on the other hand, are not necessarily rigid or flexible, but instead tend to be of higher strength. For example, spider silk, turtle carapace, and balsa wood all show strong correlations between strength (σ) and toughness (u_T_ or K_IC_). The only exception here is feather rachis; when compared with bone and bamboo, it exhibits relatively low strength across all modes of loading (see Fig 3B). In engineering design, strength and toughness are often considered mutually exclusive properties [36]. However, recent studies show that many natural materials overcome this conflict (validating our results) via hierarchical toughening mechanisms spanning from the molecular to macro-scale [37–39].

### Tensile properties of collagenous tissues

**Fig 4:**
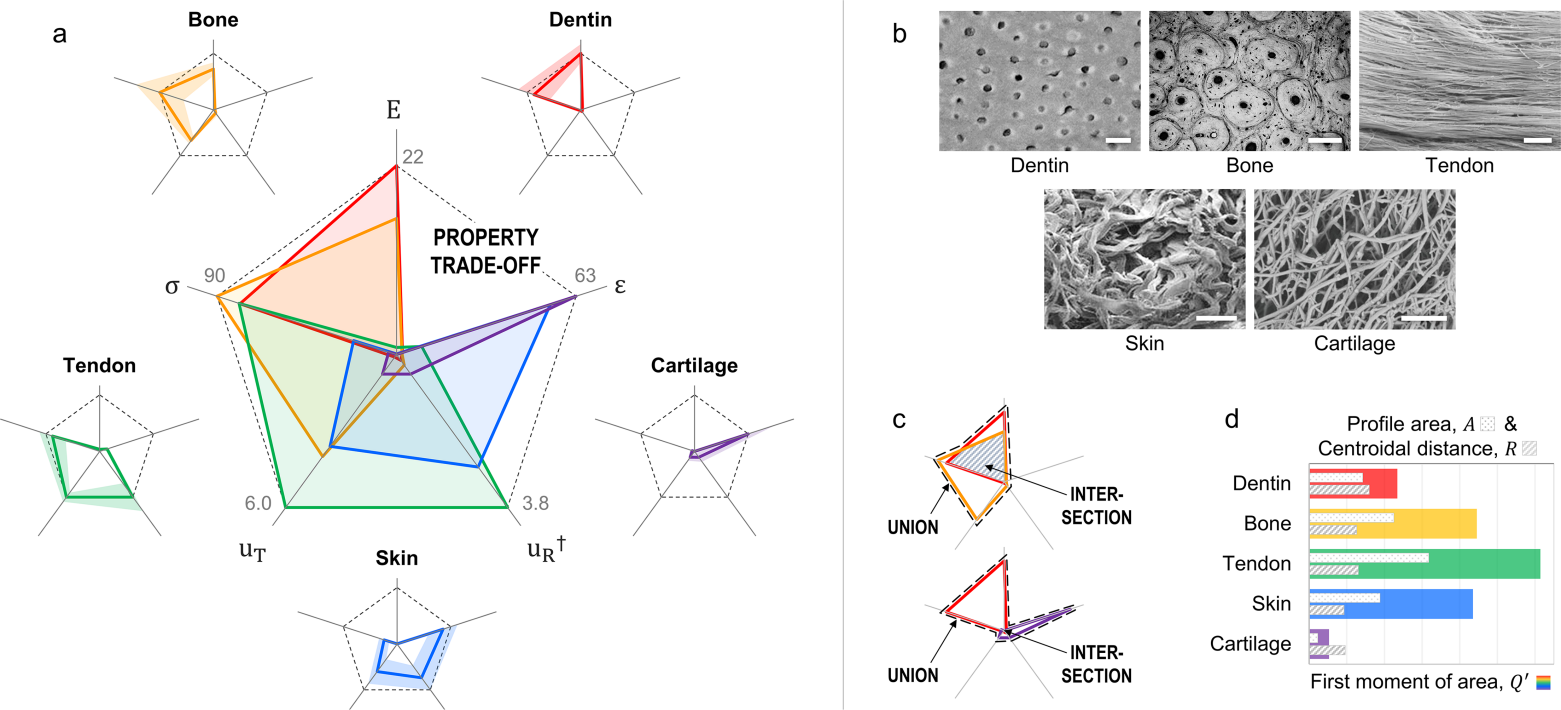
Tensile properties of dentin, bone, tendon, skin, and cartilage. (a) Normalized, permutated radar charts comparing five collagenous tissues (see Box 2), where the profile averages (lines) and standard deviations/ranges (shaded regions) are shown on the outer edges; the center plot illustrates a trade-off between stiffness (E) and extensibility (ε). (b) Micrographs illustrating key microstructural features correlating each material with its unique property profile; images adapted from literature [42–46] or provided by J. McKittrick (dentin and bone), for illustrative purposes only. Scale bars: (from left to right, top to bottom) 5 μm; 150 μm; 5 μm; 25 μm; 2 μm. (c) Radar charts showing the intersections and unions of dentin-bone (top) and dentin-cartilage (bottom). (d) Comparisons of the profile areas, *A* (dotted bars), centroidal distances, *R* (hatched bars), and relative first moments of area, *Q*′ (solid bars). Legend (units): elastic modulus, E (GPa); tensile strength, σ (MPa); toughness, u_T_ (MJ·m^-3^); resilience, u_R_^†^ (MPa); extensibility, ε (%). Notes: Data are listed in S5 Table, compiled from numerous sources (see Supporting Information); for reference, maximum values of each average property are displayed on the axes.

Dentin, bone, tendon, skin, and cartilage are collagen-based materials found in many vertebrates, and perform a diversity of tasks from skeletal support and mobility to shock absorption and protection [40, 41]. Their tensile properties are plotted in Fig 4A, illustrating the profiles occupy distinct regions of the property space correlated with form and function, as summarized in Box 2. Fig 4B shows representative microstructures of each material; for details readers are referred to the image sources [42–46].

**Box 2. Collagenous tissues. The general structure and function of five collagen-based materials: dentin, bone, tendon, skin, and cartilage (compare with Fig 4)**

***Dentin*** is ~2.2 g·cm^-3^ and composed of mineralized collagen (~70% mineral) organized into parallel arrays of microtubules; the material is relatively stiff and strong to withstand tooth bite forces [47].

***Bone*** (cortical) is ~2.0 g·cm^-3^ and composed of mineralized collagen (~65% mineral) organized into compact osteons surrounding Haversian canals (microtubules); the material is strong, yet relatively stiff and tough to provide body support and joint mobility [48].

***Tendon*** is ~1.3 g·cm^-3^ and composed of hydrated collagen (~55–70% water) organized into parallel arrays of aligned fibers; the material is tough and fairly resilient to store and transfer energy during activity [49].

***Skin*** (mammalian) is ~1.1 g·cm^-3^ and composed of hydrated collagen (~30–70% water) organized into layered networks of interwoven fibers; the material is relatively pliable (extensible, resilient, tough) to provide flexible protection [50].

***Cartilage*** (articular) is ~1.0 g∙cm^-3^ and composed of hydrated collagen (~80% water) organized into a gradient network of fibers; the material is relatively extensible to cushion joint motion [51].

Note: All tissues described above contain collagen, as well as additional minerals, proteins, or other molecules and water. For purposes of this report, only the two primary constituents are described for each material: collagen + mineral or water. For further information on these tissues, readers are referred to [40, 41, 47–51].

Notice, the sequence of properties defined in Fig 4A is similar, but not identical to Fig 3A. Although both charts compare tensile properties, our axis-sorting scheme produces different property correlations, suggesting that different functions are defined by unique property combinations. Also apparent in Fig 4A is the “gap” on the chart, whose area represents the relative intensity of a trade-off, in this case between stiffness (E) and extensibility (ε). Thus, like inferences from Fig 3, rigid and flexible tissues tend to exhibit opposing property profiles.

Another way to compare materials is by the degree of similarity between two profiles (from 0 to 1), which we measured using the Jaccard index (Eq 5) [22]. This metric suggests that dentin and bone are the most similar materials of the bunch, with the highest index of *J* = 0.440, whereas dentin and cartilage are the most dissimilar, with the lowest index of *J* = 0.003 (see Fig 4C). Interestingly, dentin and bone are dense, mineralized materials both composed of parallel arrays of microtubules (see Fig 4B), microscopic features among hierarchies of structural mechanisms that dictate their relatively rigid, damage-tolerant behaviors [40]. Conversely, dentin and cartilage have the most contrasting densities (~2.2 g·cm^-3^ vs ~1.0 g·cm^-3^) and microstructures (parallel tubule arrays vs gradient fiber networks); they also generally perform opposing functions: crushing versus cushioning [40]. Under physiological conditions (e.g., biting, support/mobility, and joint motion), axial stresses frequently develop parallel to the tubules/fibers [52–54]. However, their orientation and direction (in tension or compression) largely depend on the anatomical location, form and function of the tissues.

In addition to the Jaccard index, the first few lower-order moments (Eq 6) describe a profile’s area, centroid, and first moment of area, analogous to physical and statistical moments [20]. After exploring these and several higher-order moments, we find that the relative first moment of area about the outer limit of the property space (Eq 12) is most informative. For mechanical comparisons, the area and centroidal distance of a profile offer loose measures of performance (↑ *A* = ↑ performance) and multidimensionality (↓ *R* = ↑ multidimensionality). By this notion, a combination of the two, as in Eq 12, provides a relative approximation of multidimensional performance (from 0 to 1). As shown in Fig 4D, tendon exhibits the highest “multidimensional performance” of the group (*Q*′ = 0.25). This result makes sense because the axes of the radar chart all represent tensile properties, and tendon is the only material that predominantly carries tensile stresses. In contrast, the other four materials often support multidirectional, site-dependent normal and shear stresses.

### Feeding vs singing performance of Darwin’s finches

**Fig 5:**
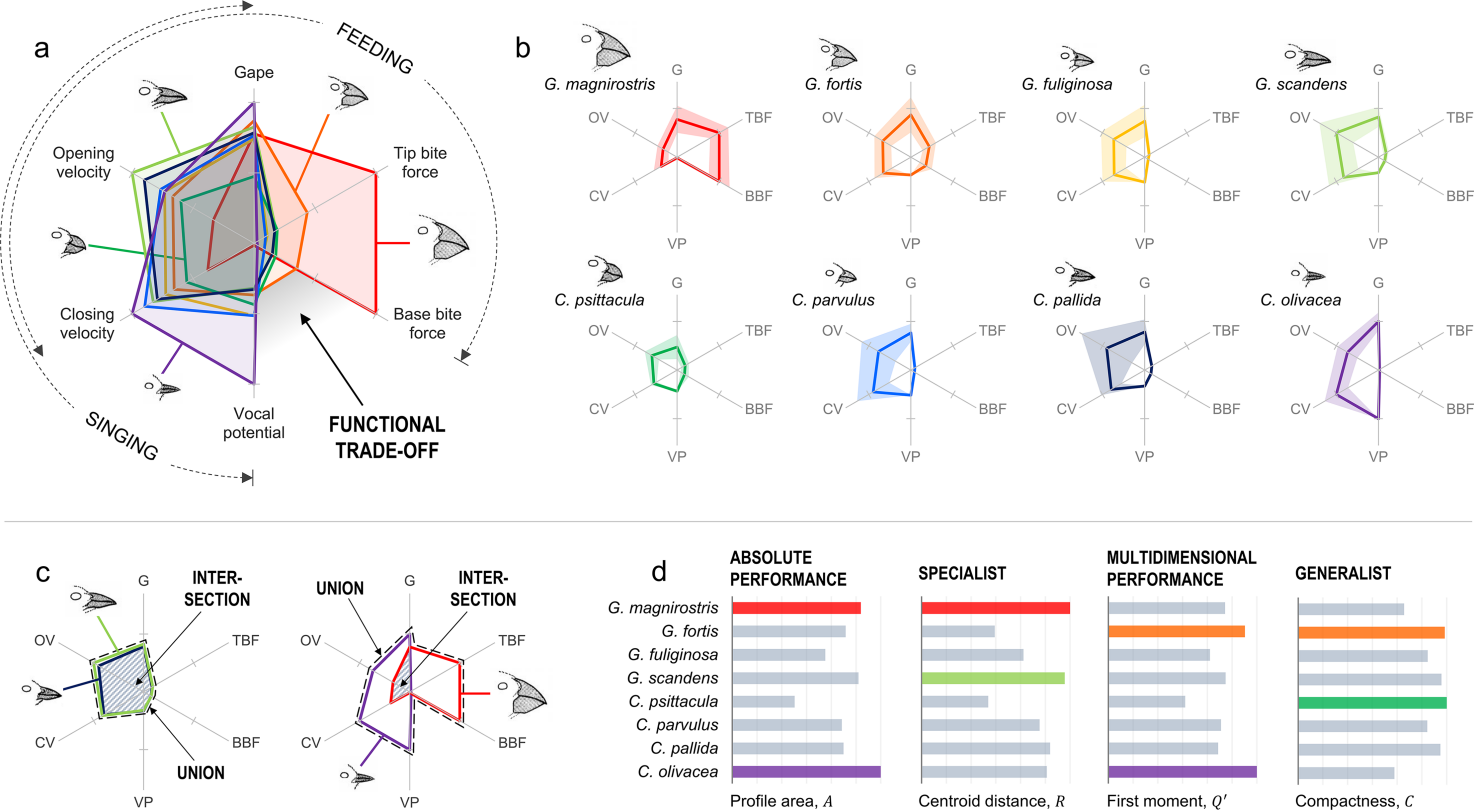
Feeding vs singing performance of Darwin’s finches. (a) Normalized, permutated radar chart comparing eight Darwin’s finches, showing the properties correlated with feeding and singing performance as well as the distinct functional trade-off between base bite force and vocal potential. (b) Individual profiles of Darwin’s finches, showing their reported averages (lines) and ranges (shaded regions). (c) Radar charts illustrating the intersections and unions of *G*. *scandens-C*. *pallida* (left) and *G*. *magnirostris-C*. *olivacea* (right). (d) Comparisons of the profile areas, *A*, centroidal distances, *R*, relative first moments of area, *Q*′, and compactness, *C*; colored bars callout the two maxima of each shape descriptor as referred in the text. Legend: base bite force (BBF), tip bite force (TBF), gape (G), opening velocity (OV), closing velocity (CV), vocal potential (VP). Notes: Beak sketches are adapted from literature [58]. Data are listed in S6 Table, compiled from numerous sources (see Supporting Information).

Beyond material comparisons, radar charts are also suitable for comparing “non-material” systems that may exhibit or require a specific balance of properties. To demonstrate, the feeding and singing performance of Darwin’s finches are shown in Fig 5A and 5B. Past studies correlate beak morphology [55] with maximum gape [56], tip and base bite forces [57], opening and closing velocities [56], and vocal potential (note: minimal vocal deviations [58] were inversed to evaluate maximal vocal potentials; see Supporting Information). The sequence of properties defined in Fig 5A supports published hypotheses [56], indicating a trade-off between feeding versus singing performance. Remarkably, close correlations exist between tip and base bite forces and between opening and closing velocities. Gape, the maximum distance between a beak’s tips, is sorted between tip bite force and opening velocity—all properties measured at the beaks’ tips [56, 57]. Vocal potential, on the other hand, is sorted next to closing velocity, suggesting that singing performance depends more on a bird’s ability to close, rather than open its beak.

In calculating the Jaccard indices of all pairs of birds, we find that *G*. *scandens* and *C*. *pallida* have the most similar profiles (*J* = 0.878), while *G*. *magnirostris* and *C*. *olivacea* have the least similar profiles (*J* = 0.097). Fig 5C shows plots of these intersections and unions. Our results agree with past reports [59, 60]: *G*. *scandens* and *C*. *pallida* have specialized, elongated beaks for probing and tool-handling [59]. In contrast, *G*. *magnirostris* and *C*. *olivacea* have the most dissimilar beak morphologies [60]; they also happen to be top performers at opposing tasks, clearly defined by the trade-off in Fig 5A between bite force (feeding) and vocal potential (singing).

Shape moment analyses (Fig 5D) suggest that *G*. *magnirostris* and *C*. *olivacea* exhibit the greatest performance (with the largest profile areas, *A*), and *G*. *magnirostris* and *G*. *scandens* are the most specialized (with the largest centroidal distances, *R*). When combined in Eq 12, values for *Q*′ suggests that *G*. *fortis* and *C*. *olivacea* exhibit the greatest multidimensional performance. However, *C*. *olivacea* exhibits the lowest bite force of the comparison, yet high performance across the other four properties related to signing performance. *G*. *fortis*, on the other hand, is not a top performer at any one property, but performs fairly well across all properties. Thus, in the case of Darwin’s finches, *Q*′ is a measure of *multidimensional* performance, and not necessarily *multifunctional* performance. Therefore, we also measured the profiles’ compactness (Eq 13). Fig 5D compares this metric, where more compact profiles are more uniformly distributed across all dimensions, and thus more multifunctional. Accordingly, *G*. *fortis* is a high-performing generalist (with relatively large *Q*′ and *C*), whereas *C*. *psittacula* is a low-performing generalist (with relatively small *Q*′, but large *C*).

## Discussion

### Limitations and possibilities

We present all property data here normalized by maxima (from 0 to 1) on radar charts of radially symmetric, equidistant axes. This constrains our analyses to *relative* performance comparisons. Like other radial projections [61–63], the scaling, rotation, addition or elimination of a radar chart’s axes could be applied to assign preference to a particular property (or set of properties). For instance, the performance of Darwin’s finches compared in Fig 5 is limited by the number and type of properties plotted as well as how closely each property describes the functions of interest: feeding versus singing. As shown in Fig 5A, we suggest feeding is described by five of the six properties, whereas singing is described by four of the six, which more heavily weights feeding over singing (5:4). One possibility to correct such bias could be accomplished by selective scaling—e.g., increasing the axis for vocal potential; another, by changing the angles between axes—e.g., rotating tip and base bite force (or opening and closing velocity) closer together, and thus increasing the angle(s) between vocal potential and the other properties. Lastly, two or more closely related properties could be collapsed into one—e.g., combining tip and base bite force (or opening and closing velocity) into, simply, bite force (or velocity). Similarly, other projection transformations [64, 65] and visual effects [66] could also help enhance the discovery and communication of new data trends, outliers, or other descriptive features.

As demonstrated, the functional significance of each shape descriptor discussed here is important to consider when using radar charts. A multitude of descriptors, including and beyond those presented [18–20], can be applied to analyze different profiles. Depending on the systems and properties being investigated, different descriptors can have different semantic meanings [18]. Additionally, algebraic combinations of two or more metrics can reveal new information. For example, *Q*′ is a simple combination of the area and centroid of a profile, *A*(1−*R*), relative to the area of the property space, *A*_*N*_. We chose this metric (Eq 12) because of its intuitive definition, where larger areas equal higher performance, and smaller centroidal distances equal higher multidimensionality (or multifunctionality in the case of functional properties). Therefore, we caution against the use of arbitrary descriptors with little to no explicable abstraction, and suggest that multiple descriptors should be compared to best understand the multidimensional mechanics of most comparative systems (as illustrated in Fig 5C and 5D).

### Extended potential

Although we restrict the present study to mechanical property data on selected biological materials and structures, we propose similar treatments of radar charts could be applied to other types of numerical or categorical data [67]. In different fields of study, the method could be used to compare nearly any collection of samples in which multiple descriptive properties can be sorted to yield an emergent effect, as in animal biomechanics [68, 69], phenotypic traits [70, 71], or multifunctional ecosystems [72, 73]. We also envision the method could be applied as a tool for biomimicry and bioinspiration [74, 75] or computational simulation and design [76, 77]. In such cases, radar charts could be used to compare alternative designs across multiple constraints or objectives, thereby directing the selection of biological systems best suited for design inspiration or guiding the invention of performance-driven materials, structures, and machines.

We also suggest using radar charts coupled with other visualization methods to augment data interpretation—e.g., coupling Figs 1 and 3. In materials selection and design, for instance, radar charts could complement traditional materials property charts (Ashby plots) [4]. That is, key property-function correlations identified using radar charts could narrow the search field of properties to be investigated via Ashby plots. In reverse, Ashby plots could narrow the search field of material classes for radar chart analyses aimed at amplifying design *multi*-functionality. In another way, structural or morphological measurements coupled with radar charts could reveal structure-property-function design rules for material systems—e.g., the fibrous microstructure of tendon is strong, tough and resilient, functioning as a robust tension tie (see Fig 4). Thus, when combined with other exploratory strategies [78, 79], radar charts show much promise across a wide range of disciplines where multidimensional datasets are ubiquitous.

## Conclusions

In this study, we present a new take on radar charts, allowing for comparative systems to be analyzed across multiple mechanical properties (*N* ≥ 3) on a single graphic. The theoretical framework behind our strategy relies on the notion that radar chart data are structured as closed polygonal profiles whose distributions provide relative measures of multidimensional performance. When permutated to yield maximal total area, the properties (axes) of a radar chart are sorted by function, which reduces the relative weight or bias of each property on performance measurements and infers task-specific correlations between properties and functions. Additionally, permutated radar charts are useful for identifying performance trade-offs, profile similarities, and other multidimensional characteristics via simple shape descriptors such as area, centroid, first moment of area, compactness, etc. [18–20]. Applying these metrics, we corroborate previous reports on the mechanics of fifteen different biological materials as well as the beaks of Darwin’s finches. We also suggest many potential applications for radar charts within the realms of biological sciences and engineering.

## Supporting information

S1 File MATLAB. The attached MATLAB code (radarchart) analyzes the collagenous tissues dataset (S5 Table), for an example. The code is used to output plots of all possible permutations, the maximal area permutation, its profile areas, centroids, relative moments, and compactness, as well as the Jaccard indices of all pairs of profiles.
(DOCX)

S1 References Additional supporting references for the six datasets (S1–S6 Tables). (DOCX)

S1 Table Tension ties. Mechanical property data are compiled from: Spider silk: major ampullate silk of *Nephila edulis* [80], frame silk of *Araneus sericatus* [81] and unspecified species [82]; Mammal tendon: collagen of adult mammalian tendon [83], human Achilles tendon [84] and rabbit Achilles tendon [85]; Mussel byssus: byssal threads of *Mytilus galloprovincialis* [86] and *Mytilus californianus* [83, 87]. Data reported as **averages** and (standard deviations) or [ranges] depending on source; data in Fig 3A displayed as normalized averages (lines) and standard deviations/ranges (shaded regions); averages calculated from minimum and maximum values of reported deviations/ranges. Properties: density (**ρ**), elastic modulus (**E**), tensile strength (**σ**_**T**_), resilience (**u**_**R**_), damping loss factor (**tan δ**), strain to failure (**ε**), toughness (**u**_**T**_).
(DOCX)

S2 Table Load-bearing beams. Mechanical property data are compiled from: Bamboo culm: trunk of *Neosinocalamus affinis* [88], internodes of *Phyllostachys pubescens* [89] and unspecified region of *Sinocalamus affinis* [90]; Cortical bone: adult human Haversian [91] and bovine femur [92, 93]; Feather rachis: flight feathers of *Larus californicus* [94] and feather keratin of *Struthio camelus* [95]. Data reported as **averages** and [ranges] depending on source; data in Fig 3B displayed as normalized averages (lines) and ranges (shaded regions); averages calculated from minimum and maximum values of reported ranges. Properties: density (**ρ**), elastic modulus (**E**), tensile strength (**σ**_**T**_), toughness (**u**_**T**_), strain to failure (**ε**), compressive strength (**σ**_**C**_), flexural strength (**σ**_**F**_).
(DOCX)

S3 Table Protective shells. Mechanical property data are compiled from: Mollusk nacre: shells of *Pinctada* [96] and *Haliotis rufescens* [97–99]; Turtle carapace: bony scutes and sutures of *Trachemys scripta elegans* [100–102], four species of turtles [103] and unspecified tortoise species [104]; Bovid horn: sheath keratin of *Ovis Canadensis* [42, 98, 105] and *Oryx gazelle* [106]. Data reported as **averages** and (standard error) or [ranges] depending on source; data in Fig 3C displayed as normalized averages (lines) and standard errors/ranges (shaded regions); averages calculated from minimum and maximum values of reported errors/ranges. Properties: density (**ρ**), elastic modulus (**E**), hardness (**H**), flexural strength (**σ**_**F**_), fracture toughness (**K**_**IC**_), strain to failure (**ε**), impact strength (**IS**).
(DOCX)

S4 Table Porous foams. Mechanical property data are compiled from: Coral skeleton: scleractinian coral, *Porites Cylindrica* [107] and several species of gorgonian corals [108]; Cancellous bone: human knees [109], human vertebrae and tibiae [110] and bovine tibiae [111]; Balsa wood: trunk of *Ochroma pyramidale* [112–114]. Data reported as **averages** and (standard deviations) or [ranges] depending on source; data in Fig 3D displayed as normalized averages (lines) and standard deviations/ranges (shaded regions); averages calculated from minimum and maximum values of reported deviations/ranges. Properties: density (**ρ**), elastic modulus (**E**), hardness (**H**), shear modulus (**G**), strain to failure (**ε**), compressive strength (**σ**_**C**_), toughness (**u**_**T**_).
(DOCX)

S5 Table Collagenous tissues. Mechanical property data are compiled from: Dentin: human teeth [52, 115], bovine teeth [116] and unspecified [117]. Bone: adult human Haversian [91] and bovine femur [92, 93]; Tendon: collagen of adult mammals [83], human Achilles [84] and rabbit Achilles [85]; Skin: human back [118] and unspecified [119]; Cartilage: porcine temporo-mandibular joint disc (TMJ) [120] and femoral articular of unspecified species [121]. Data reported as **averages** and (standard deviations) or [ranges] and {calculations} depending on source; data in Fig 4A displayed as normalized averages (lines) and standard deviations/ranges (shaded regions); averages calculated from minimum and maximum values of reported deviations/ranges. Properties: density (**ρ**), elastic modulus (**E**), tensile strength (**σ**), toughness (**u**_**T**_), and extensibility or strain to failure (**ε**); tensile resilience (**u**_**R**_) calculated by: u_R_ = σ^2^/2E.
(DOCX)

S6 Table Darwin’s finches. Feeding and singing performance averages (standard deviations) are compiled from: base and tip bite forces of male birds [57]; maximum gape, opening and closing velocity of unspecified genders [56]; vocal potentials (VP) of male birds calculated from {vocal deviations (VD)} [58] by the equation: VP_j_ = max(VD) + min(VD) − VD_j_. Data in Fig 5A and 5B displayed as normalized averages (lines) and standard deviations (shaded regions).
(DOCX)
