# Supplementary material for: Multidimensional mechanics: Performance mapping of natural biological systems using permutated radar charts
Source: PLoS One. 2018 Sep 28;13(9):e0204309. doi: 10.1371/journal.pone.0204309 (PMC6161877; doi:10.1371/journal.pone.0204309)
Supplement: S1 File — The attached MATLAB code (radarchart) analyzes the collagenous tissues dataset (S5 Table), for an example. The code is used to output plots of all possible permutations, the maximal area permutation, its profile areas, centroids, relative moments, and compactness, as well as the Jaccard indices of all pairs of profiles. (DOCX) [file pone.0204309.s001.docx]

**S1 File. MATLAB.** The attached MATLAB code (radarchart) analyzes the collagenous tissues dataset (S5 Table), for an example. The code is used to output plots of all possible permutations, the maximal area permutation, its profile areas, centroids, relative moments, and compactness, as well as the Jaccard indices of all pairs of profiles.

**Code:**

clc; % input property data from TABLE S5

close all; clear all; % P1=E, P2=sig, P3=uT, P4=uR, P5=eps

p(1,:)=[ 21.5000 78.5000 0.1000 0.1433 2.0900 ]; % dentin [P1 P2 P3 P4 P5]

p(2,:)=[ 15.5000 90.0000 4.0000 0.2613 1.8500 ]; % bone [P1 P2 P3 P4 P5]

p(3,:)=[ 0.8190 79.0000 6.0000 3.8101 8.8000 ]; % tendon [P1 P2 P3 P4 P5]

p(4,:)=[ 0.0833 21.6000 3.6000 2.8005 54.0000 ]; % skin [P1 P2 P3 P4 P5]

p(5,:)=[ 0.0164 3.9800 0.7650 0.4832 63.0500 ]; % cartilage [P1 P2 P3 P4 P5]

i=5; j=5; % i = # properties; j = # systems

An=i*1*1*sin((2*pi)/i)/2; % An = area of property space

for f=1:i

p(:,f)=p(:,f)/max(p(:,f)); % normalizes property values

theta(f)=(f-1)*((2*pi)/i); % finds angles between axes

N=perms(1:i); n=size(N); % finds all possible permutations

end

for g=1:n(1,1)

for h=1:j

P(h,1:i)=p(h,N(g,:)); % stores normalized property values

X=P(:,1:i).*cos(theta); Y=P(:,1:i).*sin(theta); % finds property coordinates (X,Y)

A(h,:)=polyarea(X(h,:),Y(h,:)); % finds profile area (A)

M(h,:)=perimeter(polyshape(X(h,:),Y(h,:))); % finds profile perimeter (M)

[XC(h,:),YC(h,:)]=centroid(polyshape(X(h,:),Y(h,:))); % finds profile centroid [XC,YC]

end

N=N; K(g,1:i)=N(g,:); % N = permutation sequence

A=A; K(g,i+1:i+j)=A; % A = profile area

R=sqrt(XC.^2+YC.^2); K(g,i+j+1:i+2*j)=R; % R = centroidal distance

Q=(A/An).*(1-R); K(g,i+2*j+1:i+3*j)=Q; % Q = first moment of area

C=2*sqrt(pi*A)./M; K(g,i+3*j+1:i+4*j)=C; % C = compactness

S=sum(A); K(g,i+4*j+1)=S; % S = total area of all profiles

for f=1:g-1

if abs(K(g,i+4*j+1)-K(f,i+4*j+1))<.000001 % removes repetitive patterns...

K(g,i+4*j+1)=0; % ...by comparing profile areas

end

end

end

k=find(K(:,i+4*j+1)); s=size(k); % finds unique permutations

K=K(k,:); K=sortrows(K,i+4*j+1); % sorts maximal area permutation last

N=K(:,1:i); NN=K(s(1,1),1:i); pr=p(:,NN); % reorders properties to max perm

Xr=pr(:,1:i).*cos(theta); Yr=pr(:,1:i).*sin(theta); % reorders X,Y coord to max perm

for g=1:j

for h=1:j

B1(:,1)=Xr(g,:); B1(:,2)=Yr(g,:); % defines X,Y coord of profile B1

B2(:,1)=Xr(h,:); B2(:,2)=Yr(h,:); % defines X,Y coord of profile B2

I=areaintersection(B1,B2,1000); % finds intersection of B1+B2

U=polyarea(Xr(g,:),Yr(g,:))+polyarea(Xr(h,:),Yr(h,:))-I; % finds union of B1+B2

J(g,h)=[I]/[U]; % finds jaccard index of B1+B2

end

end

%%%%%%%%%%%%%%%%%%%%%%%%%%%%%%%%%%%%%%%%%% plotting and data output %%%%%%%%%%%%%%%%%%%%%%%%%%%%%%%%%%%%%%%%%%%

c={[1 0 0 1];[1 .4 0 1];[0 .69 .31 1];[0 .4 1 1];[.6 0 1 1]}; % defines profile colors

Kr=K; Kr(:,i+1)=K(:,1); thetar=theta; thetar(:,i+1)=thetar(:,1); % defines circular pattern of axes...

pr=p; pr(:,i+1)=pr(:,1); % ...and property values

figure % plots all permutations - FIG 2a

for x=1:s(1,1)

subplot(3,4,x)

for y=1:j

polarplot(thetar,pr(y,Kr(x,1:i+1)),'Color',c{y},'LineWidth', 1)

hold on

ax = gca; ax.ThetaDir='clockwise';

rlim([0 1.1]); rticks([0]); rticklabels '';

thetaticks(0:360/i:360); thetaticklabels(split(sprintf('P%d ',N(x,:))));

end

end

figure % plots perm radar chart - FIG 2b

for z=1:j

polarplot(thetar,pr(z,Kr(s(1,1),1:i+1)),'Color',c{z},'LineWidth',2)

hold on

ax=gca; ax.ThetaDir='clockwise';

rlim([0 1.1]); rticks([0]); rticklabels '';

thetaticks(0:360/i:360); thetaticklabels(split(sprintf('P%d ',NN)));

end

NN=sprintf(' P%d ',transpose(NN)); % displays maximal area permutation

fprintf('Sequence of permutated radar chart = %s\n',NN);

fprintf('Total maximal area = %d\n\n',K(s(1,1),i+4*j+1))

fprintf('\t\t\t\t\tDentin\tBone\tTendon\tSkin\tCartilage\n'); % displays shape descriptors

AA=sprintf('\t%.4f',transpose(K(s(1,1),i+1:i+j)));

fprintf('Profile area, A = %s\n',AA);

RR=sprintf('\t%.4f',transpose(K(s(1,1),i+j+1:i+2*j)));

fprintf('Centroid dis, R = %s\n',RR);

QQ=sprintf('\t%.4f',transpose(K(s(1,1),i+2*j+1:i+3*j)));

fprintf(' Rel moment, Q = %s\n',QQ);

CC=sprintf('\t%.4f',transpose(K(s(1,1),i+3*j+1:i+4*j)));

fprintf(' Compactness, C = %s\n\n',CC);

fprintf('Jaccard indx, J =\n'); % displays jaccard indices

fprintf('\t\t\t\t\tDentin\tBone\tTendon\tSkin\tCartilage\n');

fprintf('\t\t\tDentin\t%.4f\t%.4f\t%.4f\t%.4f\t%.4f\n',J(1,:));

fprintf('\t\t\t Bone\t%.4f\t%.4f\t%.4f\t%.4f\t%.4f\n',J(2,:));

fprintf('\t\t\tTendon\t%.4f\t%.4f\t%.4f\t%.4f\t%.4f\n',J(3,:));

fprintf('\t\t\t Skin\t%.4f\t%.4f\t%.4f\t%.4f\t%.4f\n',J(4,:));

fprintf('\t\t Cartilage\t%.4f\t%.4f\t%.4f\t%.4f\t%.4f\n',J(5,:));

**Output:**

Sequence of permutated radar chart = P5 P4 P3 P2 P1

Total maximal area = 2.759286e+00

Dentin Bone Tendon Skin Cartilage

Profile area, A = 0.4383 0.6926 0.9777 0.5796 0.0711

Centroid dis, R = 0.4918 0.3885 0.4024 0.2864 0.2946

Rel moment, Q = 0.0937 0.1781 0.2457 0.1739 0.0211

Compactness, C = 0.7719 0.8479 0.8249 0.7937 0.4139

Jaccard indx, J =

Dentin Bone Tendon Skin Cartilage

Dentin 1.0000 0.4400 0.0178 0.0046 0.0031

Bone 0.4400 1.0000 0.2604 0.0775 0.0127

Tendon 0.0178 0.2604 1.0000 0.2795 0.0247

Skin 0.0046 0.0775 0.2795 1.0001 0.1197

Cartilage 0.0031 0.0127 0.0247 0.1197 1.0000


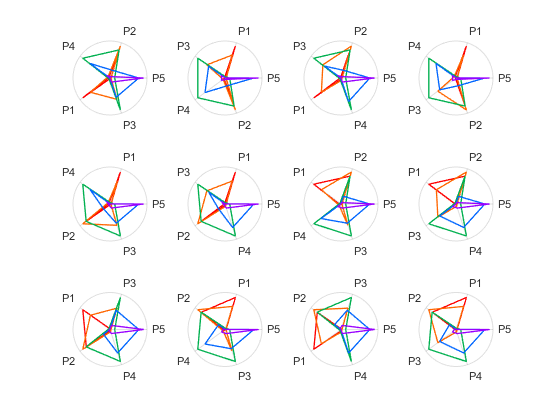
 
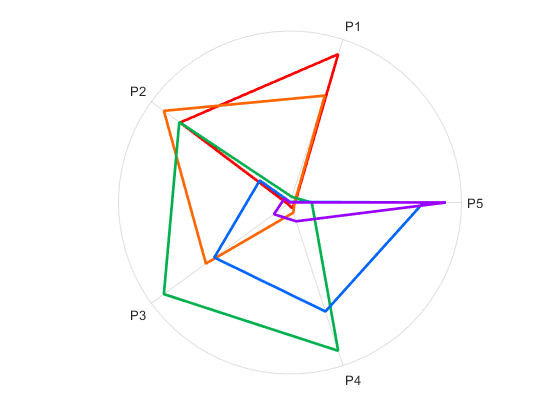


[*Published with MATLAB® R2018a*](https://www.mathworks.com/products/matlab/)
