# Supplementary material for: Multidimensional mechanics: Performance mapping of natural biological systems using permutated radar charts
Source: PLoS One. 2018 Sep 28;13(9):e0204309. doi: 10.1371/journal.pone.0204309 (PMC6161877; doi:10.1371/journal.pone.0204309)
Supplement: S1 References — (DOCX) [file pone.0204309.s002.docx]

**Supporting References**

80. Yang Y, Chen X, Shao Z, Zhou P, Porter D, Knight DP, et al. Toughness of spider silk at high and low temperatures. Advanced Materials. 2005;17(1):84-8.

81. Denny M. The physical properties of spider's silk and their role in the design of orb-webs. Journal of Experimental Biology. 1976;65(2):483-506.

82. Vollrath F, Knight DP. Liquid crystalline spinning of spider silk. Nature. 2001;410(6828):541.

83. Gosline J, Lillie M, Carrington E, Guerette P, Ortlepp C, Savage K. Elastic proteins: biological roles and mechanical properties. Philosophical Transactions of the Royal Society of London B: Biological Sciences. 2002;357(1418):121-32.

84. Wren TA, Yerby SA, Beaupré GS, Carter DR. Mechanical properties of the human achilles tendon. Clinical Biomechanics. 2001;16(3):245-51.

85. Nagasawa K, Noguchi M, Ikoma K, Kubo T. Static and dynamic biomechanical properties of the regenerating rabbit Achilles tendon. Clinical Biomechanics. 2008;23(6):832-8.

86. Waite JH, Vaccaro E, Sun C, Lucas JM. Elastomeric gradients: a hedge against stress concentration in marine holdfasts? Philosophical Transactions of the Royal Society of London B: Biological Sciences. 2002;357(1418):143-53.

87. Carrington E, Gosline JM. Mechanical design of mussel byssus: load cycle and strain rate dependence. American Malacological Bulletin. 2004;18(1/2):135-42.

88. Chen H, Cheng H, Wang G, Yu Z, Shi SQ. Tensile properties of bamboo in different sizes. Journal of Wood Science. 2015;61(6):552-61.

89. Dixon PG, Gibson LJ. The structure and mechanics of Moso bamboo material. Journal of The Royal Society Interface. 2014;11(99):20140321.

90. Low I, Che Z, Latella B. Mapping the structure, composition and mechanical properties of bamboo. Journal of Materials Research. 2006;21(8):1969-76.

91. Currey JD. The structure and mechanics of bone. Journal of Materials Science. 2012;47(1):41-54.

92. Currey JD. The design of mineralised hard tissues for their mechanical functions. Journal of Experimental Biology. 1999;202(23):3285-94.

93. Melvin JW. Fracture mechanics of bone. Journal of Biomedical Engineering. 1993;115:549-.

94. Wang B, Meyers MA. Seagull feather shaft: Correlation between structure and mechanical response. Acta Biomaterialia. 2017;48:270-88.

95. Bonser R, Saker L, Jeronimidis G. Toughness anisotropy in feather keratin. Journal of Materials Science. 2004;39(8):2895-6.

96. Jackson A, Vincent J, Turner R. Comparison of nacre with other ceramic composites. Journal of Materials Science. 1990;25(7):3173-8.

97. Barthelat F, Li C-M, Comi C, Espinosa HD. Mechanical properties of nacre constituents and their impact on mechanical performance. Journal of Materials Research. 2006;21(8):1977-86.

98. Lee S, Novitskaya EE, Reynante B, Vasquez J, Urbaniak R, Takahashi T, et al. Impact testing of structural biological materials. Materials Science and Engineering: C. 2011;31(4):730-9.

99. Wang R, Suo Z, Evans A, Yao N, Aksay I. Deformation mechanisms in nacre. Journal of Materials Research. 2001;16(9):2485-93.

100. Achrai B, Bar-On B, Wagner H. Biological armors under impact—effect of keratin coating and synthetic bio-inspired analogues. Bioinspiration & Biomimetics. 2015;10(1):016009.

101. Achrai B, Bar-On B, Wagner HD. Bending mechanics of the red-eared slider turtle carapace. Journal of the Mechanical Behavior of Biomedical Materials. 2014;30:223-33.

102. Achrai B, Wagner HD. Micro-structure and mechanical properties of the turtle carapace as a biological composite shield. Acta Biomaterialia. 2013;9(4):5890-902.

103. Magwene PM, Socha JJ. Biomechanics of turtle shells: how whole shells fail in compression. Journal of Experimental Zoology Part A: Ecological Genetics and Physiology. 2013;319(2):86-98.

104. Xu Y, Zhang L. Mechanical properties and microstructure of tortoise shell. Composites. 1995;26(4):315-8.

105. Tombolato L, Novitskaya EE, Chen P-Y, Sheppard FA, McKittrick J. Microstructure, elastic properties and deformation mechanisms of horn keratin. Acta Biomaterialia. 2010;6(2):319-30.

106. Kitchener A. Fracture toughness of horns and a reinterpretation of the horning behaviour of bovids. Journal of Zoology. 1987;213(4):621-39.

107. Shivaram A, Bose S, Bandyopadhyay A. Compressive deformation behaviour of coral Porites Cylindrica. Materials Letters. 2014;133:155-7.

108. Jeyasuria P, Lewis J. Mechanical properties of the axial skeleton in gorgonians. Coral Reefs. 1987;5(4):213-9.

109. Linde F, Hvid I, Pongsoipetch B. Energy absorptive properties of human trabecular bone specimens during axial compression. Journal of Orthopaedic Research. 1989;7(3):432-9.

110. Rho J-Y, Tsui TY, Pharr GM. Elastic properties of human cortical and trabecular lamellar bone measured by nanoindentation. Biomaterials. 1997;18(20):1325-30.

111. Ford CM, Keaveny TM. The dependence of shear failure properties of trabecular bone on apparent density and trabecular orientation. Journal of Biomechanics. 1996;29(10):1309-17.

112. Borrega M, Gibson LJ. Mechanics of balsa (Ochroma pyramidale) wood. Mechanics of Materials. 2015;84:75-90.

113. Da Silva A, Kyriakides S. Compressive response and failure of balsa wood. International Journal of Solids and Structures. 2007;44(25):8685-717.

114. Vural M, Ravichandran G. Dynamic response and energy dissipation characteristics of balsa wood: experiment and analysis. International Journal of Solids and Structures. 2003;40(9):2147-70.

115. Craig R, Peyton F. Elastic and mechanical properties of human dentin. Journal of Dental Research. 1958;37(4):710-8.

116. Gilmore R, Pollack R, Katz J. Elastic properties of bovine dentine and enamel. Archives of Oral Biology. 1970;15(8):787IN17-96.

117. Geetha M, Asokamani R, Kumar JA, Ramalingam M. Mechanical characterization of nanofiber composites. Nanofiber Composites for Biomedical Applications. 2017:117.

118. Annaidh AN, Bruyère K, Destrade M, Gilchrist MD, Otténio M. Characterization of the anisotropic mechanical properties of excised human skin. Journal of the Mechanical Behavior of Biomedical Materials. 2012;5(1):139-48.

119. Gennisson J-L, Baldeweck T, Tanter M, Catheline S, Fink M, Sandrin L, et al. Assessment of elastic parameters of human skin using dynamic elastography. IEEE Transactions on Ultrasonics, Ferroelectrics, and Frequency Control. 2004;51(8):980-9.

120. Detamore MS, Athanasiou KA. Tensile properties of the porcine temporomandibular joint disc. Journal of Biomedical Engineering. 2003;125(4):558-65.

121. Wei H-W, Sun S-S, Jao S-HE, Yeh C-R, Cheng C-K. The influence of mechanical properties of subchondral plate, femoral head and neck on dynamic stress distribution of the articular cartilage. Medical Engineering & Physics. 2005;27(4):295-304.
