# Supplementary material for: Multidimensional mechanics: Performance mapping of natural biological systems using permutated radar charts
Source: PLoS One. 2018 Sep 28;13(9):e0204309. doi: 10.1371/journal.pone.0204309 (PMC6161877; doi:10.1371/journal.pone.0204309)
Supplement: S1 Table — Mechanical property data are compiled from: Spider silk: major ampullate silk of Nephila edulis [80], frame silk of Araneus sericatus [81] and unspecified species [82]; Mammal tendon: collagen of adult mammalian tendon [83], human Achilles tendon [84] and rabbit Achilles tendon [85]; Mussel byssus: byssal threads of Mytilus galloprovincialis [86] and Mytilus californianus [83, 87]. Data reported as averages and (standard deviations) or [ranges] depending on source; data in Fig 3A displayed as normalized averages (lines) and standard deviations/ranges (shaded regions); averages calculated from minimum and maximum values of reported deviations/ranges. Properties: density (ρ), elastic modulus (E), tensile strength (σT), resilience (uR), damping loss factor (tan δ), strain to failure (ε), toughness (uT). (DOCX) [file pone.0204309.s003.docx]

**S1 Table. Tension ties.** Mechanical property data are compiled from: Spider silk: major ampullate silk of *Nephila edulis* [80], frame silk of *Araneus sericatus* [81] and unspecified species [82]; Mammal tendon: collagen of adult mammalian tendon [83], human Achilles tendon [84] and rabbit Achilles tendon [85]; Mussel byssus: byssal threads of *Mytilus galloprovincialis* [86] and *Mytilus californianus* [83, 87]. Data reported as **averages** and (standard deviations) or [ranges] depending on source; data in Fig 3a displayed as normalized averages (lines) and standard deviations/ranges (shaded regions); averages calculated from minimum and maximum values of reported deviations/ranges. Properties: density ($\boldsymbol{\rho}$), elastic modulus ($\mathbf{E}$), tensile strength ($\boldsymbol{\sigma}_{\mathbf{T}}$), resilience ($\mathbf{u}_{\mathbf{R}}$), damping loss factor ($\tan\boldsymbol{\delta}$), strain to failure ($\boldsymbol{\varepsilon}$), toughness ($\mathbf{u}_{\mathbf{T}}$).

| **TIES** | $\boldsymbol{\rho}$ | $\mathbf{E}$ | $\boldsymbol{\sigma}_{\mathbf{T}}$ | $\mathbf{u}_{\mathbf{R}}$ | $\tan\boldsymbol{\delta}$ | $\boldsymbol{\varepsilon}$ | $\mathbf{u}_{\mathbf{T}}$ |
| --- | --- | --- | --- | --- | --- | --- | --- |
|  | g·cm^-3^ | GPa | MPa | % | --- | % | MJ·m^-3^ |
| **Spider silk** ^[80-82]^ | **1.3** | **12** | **1250** | **52** | **0.05** | **33** | **137** |
|  |  | [7-16] | [500-2000] | [26-77] | [0.03-0.08] | [15-50] | [62-212] |
| **Mammal tendon** ^[83-85]^ | **1.3** | **0.8** | **79** | **90** | **0.13** | **9** | **6** |
|  |  | (0.2) | (22) | --- | [0.11-0.14] | (2) | --- |
| **Mussel byssus** ^[83, 86, 87]^ | **---** | **0.3** | **85** | **41** | **0.13** | **120** | **33** |
|  |  | [0.1-0.5] | [20-150] | [28-53] | [0.10-0.15] | [80-160] | [15-50] |
