# Supplementary material for: Multidimensional mechanics: Performance mapping of natural biological systems using permutated radar charts
Source: PLoS One. 2018 Sep 28;13(9):e0204309. doi: 10.1371/journal.pone.0204309 (PMC6161877; doi:10.1371/journal.pone.0204309)
Supplement: S2 Table — Mechanical property data are compiled from: Bamboo culm: trunk of Neosinocalamus affinis [88], internodes of Phyllostachys pubescens [89] and unspecified region of Sinocalamus affinis [90]; Cortical bone: adult human Haversian [91] and bovine femur [92, 93]; Feather rachis: flight feathers of Larus californicus [94] and feather keratin of Struthio camelus [95]. Data reported as averages and [ranges] depending on source; data in Fig 3B displayed as normalized averages (lines) and ranges (shaded regions); averages calculated from minimum and maximum values of reported ranges. Properties: density (ρ), elastic modulus (E), tensile strength (σT), toughness (uT), strain to failure (ε), compressive strength (σC), flexural strength (σF). (DOCX) [file pone.0204309.s004.docx]

**S2 Table. Load-bearing beams.** Mechanical property data are compiled from: Bamboo culm: trunk of *Neosinocalamus affinis* [88], internodes of *Phyllostachys pubescens* [89] and unspecified region of *Sinocalamus affinis* [90]; Cortical bone: adult human Haversian [91] and bovine femur [92, 93]; Feather rachis: flight feathers of *Larus californicus* [94] and feather keratin of *Struthio camelus* [95]. Data reported as **averages** and [ranges] depending on source; data in Fig 3b displayed as normalized averages (lines) and ranges (shaded regions); averages calculated from minimum and maximum values of reported ranges. Properties: density ($\boldsymbol{\rho}$), elastic modulus ($\mathbf{E}$), tensile strength ($\boldsymbol{\sigma}_{\mathbf{T}}$), toughness ($\mathbf{u}_{\mathbf{T}}$), strain to failure ($\boldsymbol{\varepsilon}$), compressive strength ($\boldsymbol{\sigma}_{\mathbf{C}}$), flexural strength ($\boldsymbol{\sigma}_{\mathbf{F}}$).

| **BEAMS** | $\boldsymbol{\rho}$ | $\mathbf{E}$ | $\boldsymbol{\sigma}_{\mathbf{T}}$ | $\mathbf{u}_{\mathbf{T}}$ | $\boldsymbol{\varepsilon}$ | $\boldsymbol{\sigma}_{\mathbf{C}}$ | $\boldsymbol{\sigma}_{\mathbf{F}}$ |
| --- | --- | --- | --- | --- | --- | --- | --- |
|  | g·cm^-3^ | GPa | MPa | kJ·m^-2^ | % | MPa | MPa |
| **Bamboo culm** ^[88-90]^ | **0.6** | **20** | **450** | **2*** | **2** | **69** | **164** |
|  |  | [17-24] | [290-610] | [2-3] | [2-3] | --- | [126-203] |
| **Cortical bone** ^[91-93]^ | **2.1** | **16** | **90** | **2** | **2** | **165** | **210** |
|  |  | [13-18] | [50-130] | [1-3] | [1-3] | [130-200] | [200-220] |
| **Feather rachis** ^[94, 95]^ | **---** | **4** | **166** | **11** | **10** | **28** | **91** |
|  |  | [3-4] | [88-244] | [3-18] | [6-14] | [1-54] | [69-113] |

* fracture toughness [90] converted from MPa·m^1/2^ to kJ·m^-2^ by the equation: $u_{T}=K_{\mathrm{IC}}^{2}/E$
