# Supplementary material for: Multidimensional mechanics: Performance mapping of natural biological systems using permutated radar charts
Source: PLoS One. 2018 Sep 28;13(9):e0204309. doi: 10.1371/journal.pone.0204309 (PMC6161877; doi:10.1371/journal.pone.0204309)
Supplement: S3 Table — Mechanical property data are compiled from: Mollusk nacre: shells of Pinctada [96] and Haliotis rufescens [97–99]; Turtle carapace: bony scutes and sutures of Trachemys scripta elegans [100–102], four species of turtles [103] and unspecified tortoise species [104]; Bovid horn: sheath keratin of Ovis Canadensis [42, 98, 105] and Oryx gazelle [106]. Data reported as averages and (standard error) or [ranges] depending on source; data in Fig 3C displayed as normalized averages (lines) and standard errors/ranges (shaded regions); averages calculated from minimum and maximum values of reported errors/ranges. Properties: density (ρ), elastic modulus (E), hardness (H), flexural strength (σF), fracture toughness (KIC), strain to failure (ε), impact strength (IS). (DOCX) [file pone.0204309.s005.docx]

**S3 Table. Protective shells.** Mechanical property data are compiled from: Mollusk nacre: shells of *Pinctada* [96] and *Haliotis rufescens* [97-99]; Turtle carapace: bony scutes and sutures of *Trachemys scripta elegans* [100-102], four species of turtles [103] and unspecified tortoise species [104]; Bovid horn: sheath keratin of *Ovis Canadensis* [42, 98, 105] and *Oryx gazelle* [106]. Data reported as **averages** and (standard error) or [ranges] depending on source; data in Fig 3c displayed as normalized averages (lines) and standard errors/ranges (shaded regions); averages calculated from minimum and maximum values of reported errors/ranges. Properties: density ($\boldsymbol{\rho}$), elastic modulus ($\mathbf{E}$), hardness ($\mathbf{H}$), flexural strength ($\boldsymbol{\sigma}_{\mathbf{F}}$), fracture toughness ($\mathbf{K}_{\mathbf{IC}}$), strain to failure ($\boldsymbol{\varepsilon}$), impact strength ($\mathbf{IS}$).

| **SHELLS** | $\boldsymbol{\rho}$ | $\mathbf{E}$ | $\mathbf{H}$ | $\boldsymbol{\sigma}_{\mathbf{F}}$ | $\mathbf{K}_{\mathbf{IC}}$ | $\boldsymbol{\varepsilon}$ | $\mathbf{IS}$ |
| --- | --- | --- | --- | --- | --- | --- | --- |
|  | g·cm^-3^ | GPa | MPa | MPa | MPa·m^1/2^ | % | kJ·m^-2^ |
| **Mollusk nacre** ^[96-99]^ | **2.7** | **68** | **1500** | **209** | **4** | **0.8** | **12** |
|  |  | [66-69] | [500-2500] | [194-223] | [2-5] | [0.6-1.0] | --- |
| **Turtle carapace** ^[100-104]^ | **---** | **6** | **339** | **246** | **36** | **10** | **15** |
|  |  | [2-11] | [7-670] | [114-378] | --- | [3-18] | [7-22] |
| **Bovid horn** ^[42, 98, 105, 106]^ | **1.2** | **2** | **145** | **83** | **5** | **14** | **71** |
|  |  | [1-2] | --- | [39-127] | (1) | --- | --- |
