# Supplementary material for: Multidimensional mechanics: Performance mapping of natural biological systems using permutated radar charts
Source: PLoS One. 2018 Sep 28;13(9):e0204309. doi: 10.1371/journal.pone.0204309 (PMC6161877; doi:10.1371/journal.pone.0204309)
Supplement: S4 Table — Mechanical property data are compiled from: Coral skeleton: scleractinian coral, Porites Cylindrica [107] and several species of gorgonian corals [108]; Cancellous bone: human knees [109], human vertebrae and tibiae [110] and bovine tibiae [111]; Balsa wood: trunk of Ochroma pyramidale [112–114]. Data reported as averages and (standard deviations) or [ranges] depending on source; data in Fig 3D displayed as normalized averages (lines) and standard deviations/ranges (shaded regions); averages calculated from minimum and maximum values of reported deviations/ranges. Properties: density (ρ), elastic modulus (E), hardness (H), shear modulus (G), strain to failure (ε), compressive strength (σC), toughness (uT). (DOCX) [file pone.0204309.s006.docx]

**S4 Table. Porous foams.** Mechanical property data are compiled from: Coral skeleton: scleractinian coral, *Porites Cylindrica* [107] and several species of gorgonian corals [108]; Cancellous bone: human knees [109], human vertebrae and tibiae [110] and bovine tibiae [111]; Balsa wood: trunk of *Ochroma pyramidale* [112-114]. Data reported as **averages** and (standard deviations) or [ranges] depending on source; data in Fig 3d displayed as normalized averages (lines) and standard deviations/ranges (shaded regions); averages calculated from minimum and maximum values of reported deviations/ranges. Properties: density ($\boldsymbol{\rho}$), elastic modulus ($\mathbf{E}$), hardness ($\mathbf{H}$), shear modulus ($\mathbf{G}$), strain to failure ($\boldsymbol{\varepsilon}$), compressive strength ($\boldsymbol{\sigma}_{\mathbf{C}}$), toughness ($\mathbf{u}_{\mathbf{T}}$).

| **FOAMS** | $\boldsymbol{\rho}$ | $\mathbf{E}$ | $\mathbf{H}$ | $\mathbf{G}$ | $\boldsymbol{\varepsilon}$ | $\boldsymbol{\sigma}_{\mathbf{C}}$ | $\mathbf{u}_{\mathbf{T}}$ |
| --- | --- | --- | --- | --- | --- | --- | --- |
|  | g·cm^-3^ | GPa | MPa | GPa | % | MPa | MJ·m^-3^ |
| **Coral skeleton** ^[107, 108]^ | **---** | **12** | **2207**^‡^ | **0.5**^†^ | **0.9** | **9** | **0** |
|  |  | (2) | (196) | [0.1-0.9] | --- | (1) | --- |
| **Cancellous bone** ^[109-111]^ | **0.4** | **0.6** | **426** | **0.3** | **2.2** | **7** | **0.1** |
|  |  | [0.1-1.2] | [262-568] | [0.1-0.5] | [1.2-3.3] | [1-14] | [0-0.2] |
| **Balsa wood** ^[112-114]^ | **0.2** | **3** | **570** | **0.2** | **1.0** | **23** | **13.5** |
|  |  | [0-7] | (110) | [0-0.4] | --- | [6-39] | [2-25] |

^†^ reported measurements for torsion moduli of gorgonian corals [108].

^‡^ Vickers microhardness of scleractinian corals [107] converted from HV_0.1_ to MPa by the equation: $H=9.81 \times\mathrm{HV}$
