# Supplementary material for: Multidimensional mechanics: Performance mapping of natural biological systems using permutated radar charts
Source: PLoS One. 2018 Sep 28;13(9):e0204309. doi: 10.1371/journal.pone.0204309 (PMC6161877; doi:10.1371/journal.pone.0204309)
Supplement: S5 Table — Mechanical property data are compiled from: Dentin: human teeth [52, 115], bovine teeth [116] and unspecified [117]. Bone: adult human Haversian [91] and bovine femur [92, 93]; Tendon: collagen of adult mammals [83], human Achilles [84] and rabbit Achilles [85]; Skin: human back [118] and unspecified [119]; Cartilage: porcine temporo-mandibular joint disc (TMJ) [120] and femoral articular of unspecified species [121]. Data reported as averages and (standard deviations) or [ranges] and {calculations} depending on source; data in Fig 4A displayed as normalized averages (lines) and standard deviations/ranges (shaded regions); averages calculated from minimum and maximum values of reported deviations/ranges. Properties: density (ρ), elastic modulus (E), tensile strength (σ), toughness (uT), and extensibility or strain to failure (ε); tensile resilience (uR) calculated by: uR = σ2/2E. (DOCX) [file pone.0204309.s007.docx]

**S5 Table. Collagenous tissues.** Mechanical property data are compiled from: Dentin: human teeth [52, 115], bovine teeth [116] and unspecified [117]. Bone: adult human Haversian [91] and bovine femur [92, 93]; Tendon: collagen of adult mammals [83], human Achilles [84] and rabbit Achilles [85]; Skin: human back [118] and unspecified [119]; Cartilage: porcine temporo-mandibular joint disc (TMJ) [120] and femoral articular of unspecified species [121]. Data reported as **averages** and (standard deviations) or [ranges] and {calculations} depending on source; data in Fig 4a displayed as normalized averages (lines) and standard deviations/ranges (shaded regions); averages calculated from minimum and maximum values of reported deviations/ranges. Properties: density ($\boldsymbol{\rho}$), elastic modulus ($\mathbf{E}$), tensile strength ($\boldsymbol{\sigma}$), toughness ($\mathbf{u}_{\mathbf{T}}$), and extensibility or strain to failure ($\boldsymbol{\varepsilon}$); tensile resilience ($\mathbf{u}_{\mathbf{R}}$) calculated by: $u_{R}=\sigma^{2}/2E$.

| **COLLAGENS** | $\boldsymbol{\rho}$ | $\mathbf{E}$ | $\boldsymbol{\sigma}$ | $\mathbf{u}_{\mathbf{T}}$ | $\mathbf{u}_{\mathbf{R}}$ | $\boldsymbol{\varepsilon}$ |
| --- | --- | --- | --- | --- | --- | --- |
|  | g·cm^-3^ | GPa | MPa | MJ·m^-3^ | MPa | % |
| **Dentin** ^[52, 115-117]^ | **2.2** | **22** | **79** | **0.1** | {0.1} | **2** |
|  |  | [18-25] | [52-105] | --- |  | --- |
| **Bone** ^[91-93]^ | **2.1** | **16** | **90** | **4** | {0.3} | **2** |
|  |  | [13-18] | [50-130] | --- |  | [1-3] |
| **Tendon** ^[83-85]^ | **1.3** | **0.8** | **79** | **6** | {3.8} | **9** |
|  |  | (0.2) | (22) | --- |  | (2) |
| **Skin** ^[118, 119]^ | **1.1** | **0.08** | **22** | **3.6** | {2.8} | **54** |
|  |  | (0.03) | (8) | (1.6) |  | (17) |
| **Cartilage** ^[120, 121]^ | **1.0** | **0.02** | **4** | **0.8** | {0.5} | **63** |
|  |  | [0-0.03] | [1-7] | [0.3-1.2] |  | [31-95] |
