# Supplementary material for: Multidimensional mechanics: Performance mapping of natural biological systems using permutated radar charts
Source: PLoS One. 2018 Sep 28;13(9):e0204309. doi: 10.1371/journal.pone.0204309 (PMC6161877; doi:10.1371/journal.pone.0204309)
Supplement: S6 Table — Feeding and singing performance averages (standard deviations) are compiled from: base and tip bite forces of male birds [57]; maximum gape, opening and closing velocity of unspecified genders [56]; vocal potentials (VP) of male birds calculated from {vocal deviations (VD)} [58] by the equation: VPj = max(VD) + min(VD) − VDj. Data in Fig 5A and 5B displayed as normalized averages (lines) and standard deviations (shaded regions). (DOCX) [file pone.0204309.s008.docx]

**S6 Table.** **Darwin’s finches.** Feeding and singing performance averages (standard deviations) are compiled from: base and tip bite forces of male birds [57]; maximum gape, opening and closing velocity of unspecified genders [56]; vocal potentials ($\mathrm{VP}$) of male birds calculated from {vocal deviations ($\mathrm{VD}$)} [58] by the equation: $\mathrm{VP}_{j}=\max\left( \mathrm{VD} \right)+\min\left( \mathrm{VD} \right)-VD_{j}$. Data in Fig 5a-b displayed as normalized averages (lines) and standard deviations (shaded regions).

| **FINCHES** | **Base bite force** | **Tip bite force** | **Maximum Gape** | **Opening velocity** | **Closing velocity** | **Vocal potential** |
| --- | --- | --- | --- | --- | --- | --- |
|  | N | N | mm | m·s^-1^ | m·s^-1^ |  |
| ***G. magnirostris*** | 99.6 (24.4) | 64.8 (14.0) | 9.29 (3.32) | 0.10 (0.04) | 0.11 (0.05) | 0.05 {3.54} |
| ***G. fortis*** | 35.2 (14.2) | 28.3 (10.2) | 10.41 (4.31) | 0.20 (0.06) | 0.19 (0.06) | 1.30 {2.29} |
| ***G. fuliginosa*** | 8.3 (2.8) | 7.0 (2.4) | 8.87 (3.96) | 0.22 (0.09) | 0.21 (0.08) | 1.82 {1.77} |
| ***G. scandens*** | 15.0 (5.2) | 11.7 (4.0) | 9.86 (2.45) | 0.30 (0.12) | 0.24 (0.10) | 1.13 {2.46} |
| ***C. psittacula*** | 17.8 (9.5) | 12.3 (5.9) | 5.68 (2.95) | 0.18 (0.05) | 0.16 (0.02) | 1.54 {2.05} |
| ***C. parvulus*** | 8.4 (1.1) | 6.1 (1.4) | 9.15 (2.19) | 0.23 (0.12) | 0.26 (0.11) | 1.82 {1.77} |
| ***C. pallida*** | 15.0 (2.4) | 10.9 (1.9) | 9.38 (3.04) | 0.27 (0.18) | 0.23 (0.07) | 1.16 {2.43} |
| ***C. olivacea*** | 2.8 (0.6) | 1.6 (0.4) | 11.95 (2.16) | 0.22 (0.07) | 0.29 (0.08) | 3.54 {0.05} |
